# Non-invasive Follicular Thyroid Neoplasm with Papillary-like Nuclear Features and *CREB3L2::PPARγ* Fusion

**DOI:** 10.1007/s12105-025-01837-4

**Published:** 2025-08-21

**Authors:** Christopher J. Dennis, Prokopios P. Argyris, Nicole A. Cipriani

**Affiliations:** Department of Pathology, The University of Chicago Medicine, 5841 S. Maryland Avenue, MC 6101, Chicago, IL 60637 USA

**Keywords:** NIFTP, RAS-like mutation, CREB3L2:PPAR*γ*, Follicular thyroid neoplasm, Intranuclear cytoplasmic pseudoinclusions, Ubiquitin

## Abstract

A 58-year-old woman presented with an asymptomatic, soft-to-palpation, mobile, right thyroid mass. Subsequent ultrasonography revealed numerous, bilateral, mildly heterogenous, cystic and solid nodules, including a hypoechoic, 1.7 × 1.0 × 1.6 cm, solid, left superior pole nodule of intermediate morphologic suspicion, meeting ATA criteria for FNA biopsy. Cytopathologic diagnosis was suspicious for papillary thyroid carcinoma and ThyroSeq molecular testing revealed an underlying *CREB3L2::PPARγ* fusion, implying a high risk for “malignancy or non-invasive follicular thyroid neoplasm with papillary-like nuclear features (NIFTP)”. A left thyroid lobectomy was performed. Gross examination revealed a 1.1 × 0.7 × 1.0 cm, white-tan, firm, well-circumscribed nodule lacking gross evidence of invasion, which was entirely submitted for histologic examination. Microscopically, the nodule was fully enveloped by a thin fibrous capsule and composed entirely of microfollicles. Follicular epithelial cells featured moderate amounts of pale, eosinophilic, cytoplasm, and enlarged, wrinkled or irregular nuclei with chromatin clearing, grooves, and occasional intranuclear cytoplasmic pseudoinclusions. Papillary or solid growth patterns, psammoma bodies, increased mitoses, necrosis, or invasion were absent. As anticipated, immunostaining using the BRAF V600E mutant specific antibody (VE1) was negative. However, the intranuclear pseudoinclusions were negative for ubiquitin. Here, we report the clinical, radiologic, cytologic, histologic, and molecular characteristics of an example of NIFTP harboring a rare *CREB3L2::PPARγ* fusion.

Molecular advances have revolutionized our understanding of the mutational landscape of thyroid neoplasia. According to the 5th Edition WHO classification [1], NIFTP represents a non-invasive, encapsulated or well-demarcated, follicular cell-derived tumor defined by a follicular growth pattern and nuclei that have some features characteristically attributed to PTC. NIFTP is categorized as a “low-risk” neoplasm, implying an extremely low risk of metastasis, and most cases are driven by *RAS*-like genetic abnormalities, chiefly *NRAS* mutations followed by *HRAS* and *KRAS* [1, 2].

A 58-year-old woman with an otherwise non-contributory medical history presented for evaluation of an asymptomatic, soft-to-palpation, mobile, right thyroid mass. Subsequent ultrasonography revealed numerous, bilateral, mildly heterogenous, cystic and solid nodules, including a hypoechoic, 1.7 × 1.0 × 1.6 cm, solid, left superior pole nodule of intermediate morphologic suspicion, meeting ATA criteria for FNA biopsy recommendation (Fig. 1A).

**Fig. 1:**
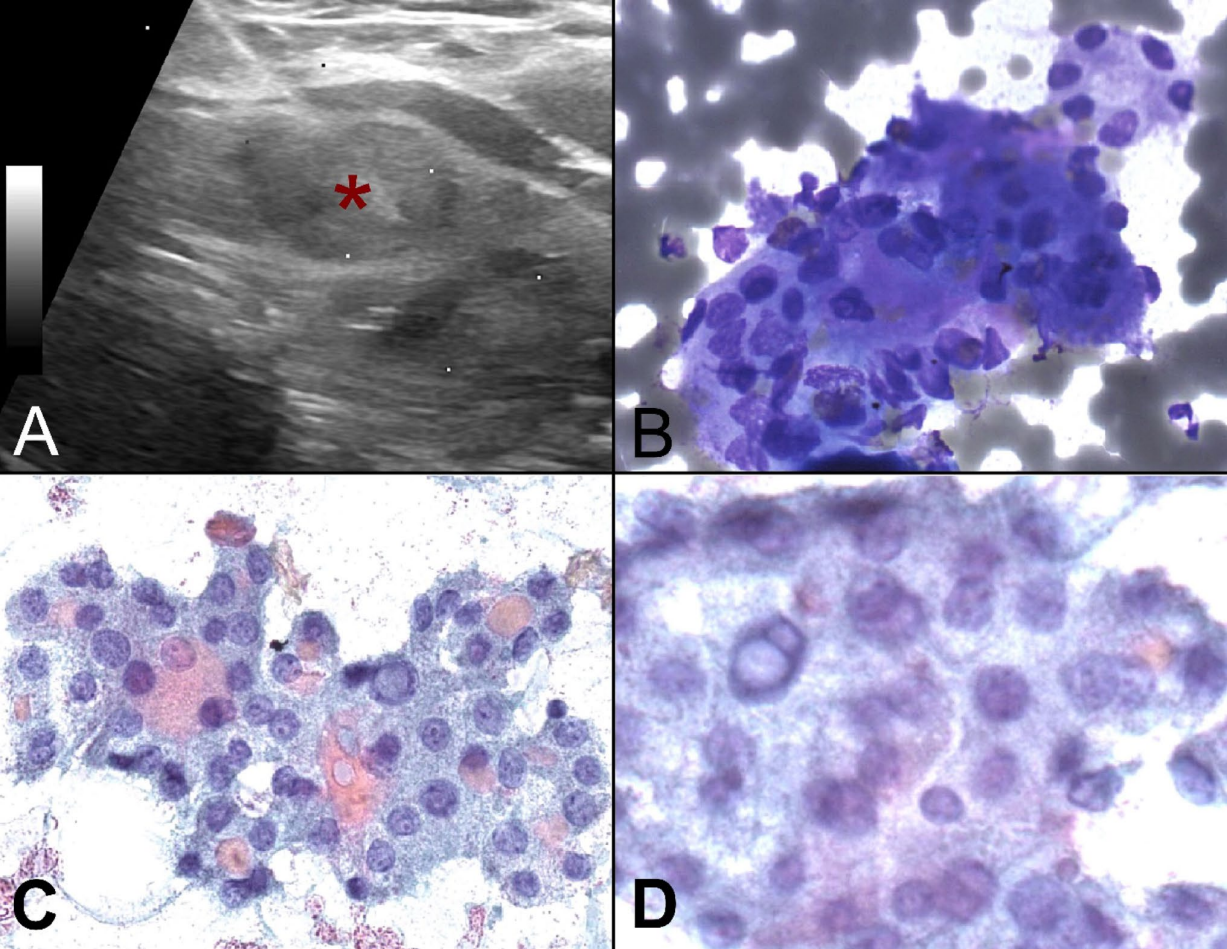
(**A**) Ultrasonography showing a hypoechoic, 1.7 x 1.0 x 1.6 cm, solid, nodule of the left superior pole (asterisk); (**B**) Diff Quik-stained smears showing solid clusters of epithelial cells with a focal microfollicular pattern; (**C**) and (**D**) Pap-stained smears highlighting lesional cells with enlarged, rounded nuclei, rare grooves and occasional intranuclear cytoplasmic pseudoinclusions, organized in microfollicles containing colloid

An FNA biopsy revealed crowded collections of cells with focal microfollicular pattern (Fig. 1B, C) centrally featuring colloid material (Fig. 1C). Lesional cells exhibited enlarged, predominantly round nuclei, with rare grooves and occasional intranuclear cytoplasmic pseudoinclusions (Fig. 1C, D). Papillae and psammoma bodies were absent. Cytopathologic diagnosis was suspicious for papillary thyroid carcinoma (PTC) and material was sent for ThyroSeq (Sonic Healthcare USA, CBLPath Laboratories, Rye Brook, NY) molecular testing, which revealed a *CREB3L2::PPARγ* rearrangement, implying a high risk for “malignancy or non-invasive follicular thyroid neoplasm with papillary-like nuclear features (NIFTP)”. A left thyroid lobectomy was performed.

Gross examination revealed a 1.1 × 0.7 × 1.0 cm, white-tan, firm, well-circumscribed nodule lacking gross evidence of invasion (Fig. 2A). The nodule was entirely submitted for histologic examination. Microscopically, the nodule was fully enveloped by a thin fibrous capsule (Fig. 2B, C) and composed entirely of microfollicular structures (Fig. 2D). Microfollicles comprised epithelial cells featuring moderate amounts of pale, eosinophilic, cytoplasm and enlarged nuclei with chromatin clearing (Fig. 3A, B), wrinkled, irregular contours, grooves, and occasional intranuclear cytoplasmic pseudoinclusions (Fig. 3B, B insets, 3C). Papillary or solid growth patterns, psammoma bodies, increased mitoses, necrosis, or invasion were not observed. Immunohistochemical stain using the BRAF V600E mutant specific antibody (VE1) was negative (Fig. 3D), unsurprising given the underlying *CREB3L2::PPARγ* fusion discovered on FNA. Intranuclear pseudoinclusions were also negative for ubiquitin expression (Fig. 3D inset). Collectively, the cytologic, histologic, immunophenotypic, and molecular findings supported a diagnosis of NIFTP.

**Fig. 2:**
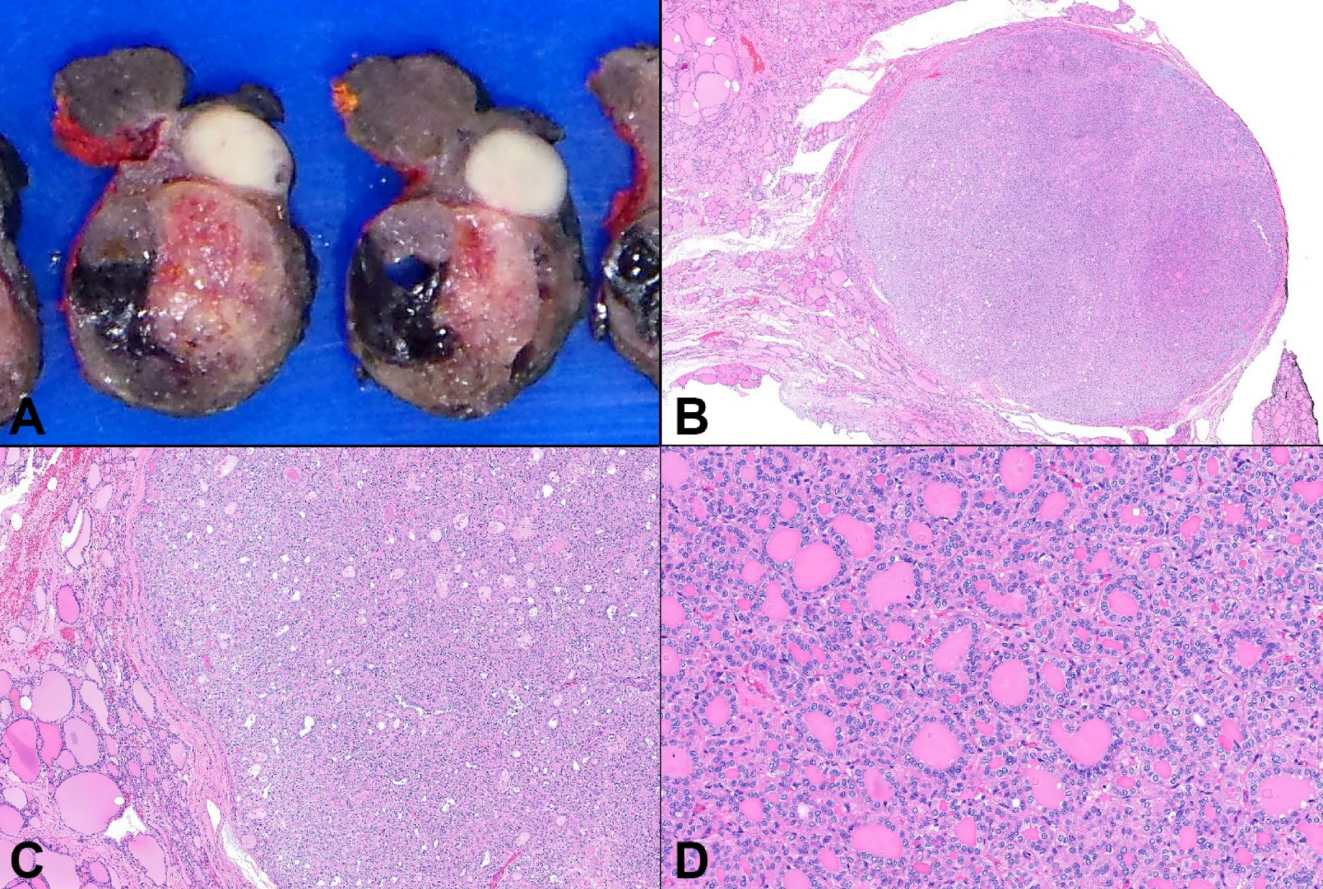
(**A**) Macroscopic examination of the left thyroid lobectomy specimen depicting a 1.1 x 0.7 x 1.0 cm, white-tan, firm, well-circumscribed nodule lacking gross evidence of invasion; (**B**) and (**C**) Low-power photomicrographs revealing a nodule fully enveloped by a thin fibrous capsule; (**D**) The lesion is composed entirely of microfollicular structures (H&E stain; original magnification B: 1x, C: 6.5x, and D: 22x)

**Fig. 3:**
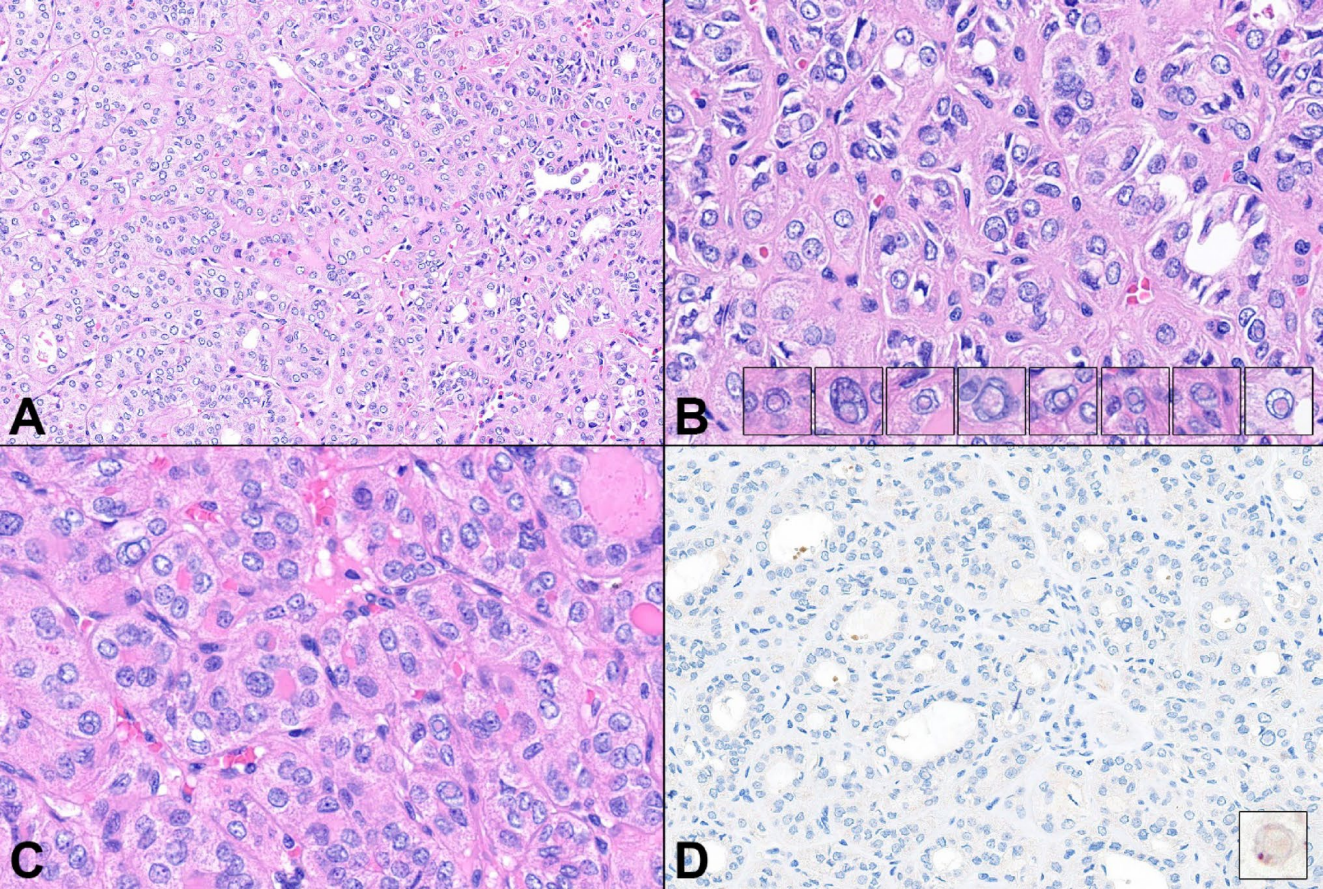
(**A**–**C**) Medium- and high-power photomicrographs displaying epithelial cells arranged in a microfollicular growth pattern featuring moderate amounts of pale, eosinophilic, cytoplasm, and enlarged, wrinkled and irregular nuclei with chromatin clearing, grooves, and occasional intranuclear cytoplasmic pseudoinclusions (insets) (H&E stain; original magnification A: 22x, B and C: 40x); (**D**) Lesional cells are negative for BRAF V600E using the mutant specific antibody (VE1), while intranuclear cytoplasmic pseudoinclusions are negative for ubiquitin by immunohistochemistry (inset) (original magnification D: 25x)

Genetic aberrations involving *PPARγ*, *THADA*, and *EIF1AX* are uncommon accounting for merely 6–17% of NIFTPs [3]. Notably, *PPARγ* fusions, primarily *PAX8::PPARγ*, are observed in 25–45% of follicular-patterned thyroid neoplasms, whereas *CREB3L2::PPARγ* is exceedingly rare and present in less than 3% of such tumors [4]. The *CREB3L2::PPARγ* fusion product induces tumorigenesis by markedly stimulating thyroid cell proliferation and growth [4].

This tumor is unique because it harbors a rare genetic driver as well as intranuclear cytoplasmic pseudoinclusions, which are also considered to be quite infrequent in NIFTP. Ubiquitin is frequently expressed within the cytoplasm of pseudoinclusions in classic PTC but not pseudo-pseudoinclusions of NIFTP [5]. In this example, despite the H&E morphology being characteristic for true pseudoinclusions, ubiquitin is absent, raising the possibility of a difference in the way these structures are formed. Is this phenomenon a morphologic idiosyncrasy or a true genomic or epigenomic difference in subcellular structure? While this report cannot answer this question, it presents a morphologic peculiarity together with a rare *CREB3L2::PPARγ* fusion in a follicular neoplasm that is currently regarded as NIFTP.

## Data Availability

No datasets were generated or analysed during the current study.
